# The bud awakens: Interplay among hormones and sugar controls bud release

**DOI:** 10.1093/plphys/kiad165

**Published:** 2023-03-21

**Authors:** Sebastian R Moreno

**Affiliations:** Assistant Features Editor, Plant Physiology, American Society of Plant Biologists, USA; Sainsbury Laboratory, University of Cambridge, Cambridge CB2 1LR, UK

Shoot branching is coordinated by endogenous and environmental signals controlling the growth of dormant buds along the stem. The development of axillary buds is inhibited by signals from the shoot apex, a phenomenon called apical dominance (Snow 1925). Thimman and Skoog's (1933) classic experiment uncovered auxin as the hormone behind apical dominance. Cytokinin (CK) and strigolactones (SLs) have also been implicated in the hormonal regulation of bud outgrowth: CK promotes and SLs inhibit bud outgrowth (Domagalska and Leyser 2011; Tanaka et al. 2006). Although the role of auxin in maintaining axillary bud growth is well established, the exact signal triggering bud release remains controversial. Some studies suggest sucrose as the molecule responsible for releasing dormant buds and show a poor association between the initial bud growth and auxin levels after decapitation (Chabikwa et al. 2019; Mason et al. 2014). Although many signals have been associated with bud growth, how these different hormones and nutrients are interconnected to modulate bud growth is still not fully understood.

In this issue of *Plant Physiology*, Cao et al. (2023) provide evidence supporting the role of sucrose as the signal that triggers changes in CK, auxin, and gibberellin (GA) concentrations to modulate bud outgrowth and to sustain bud growth. The researchers observed that in pea (*Pisum sativum*) buds outside the auxin-depletion region after decapitation, bud release is triggered by sugar-elicited changes in CK levels with the subsequent downregulation of the inhibitory role of SL (Figure). In internodes close to the apex, the authors observed that auxin levels decreased while CK levels increased, as has been previously reported (Tanaka et al. 2006; van Rongen et al. 2019). On the contrary, in internodes and buds outside the auxin-depletion zone, the inverse association between CK and auxin was disrupted with changes in CK levels occurring one hour after decapitation, while changes in auxin were observed in buds only after 3 h. Treatments with the synthetic auxin NAA applied to the decapitated stump did not affect either the decapitation-induced accumulation of CK levels or the expression of CK biosynthesis genes, suggesting that the enhanced CK levels in lower internodes are unlikely triggered by the depletion of auxin.

Since sucrose and trehalose 6-phosphate (Tre6P) release the dormancy of axillary buds in decapitated plants (Mason et al. 2014), the authors explored whether sucrose is also responsible for the enhanced CK levels in buds after apex decapitation. The researchers found that supplying sucrose to the excised stems was sufficient to release bud dormancy and increase endogenous CK levels.

Unlike CK, application of SL on axillary buds represses bud outgrowth (Gomez-Roldan et al. 2008). However, the crosstalk between SL and CK behind bud outgrowth is not completely understood. In this paper, through quantifying gene expression of several CK-related genes, the authors nicely demonstrated that SL inhibits CK levels by increasing CK degradation and decreasing CK biosynthesis. In addition, the authors quantified bud growth after treating with synthetic CK BAP and GR24 (synthetic SL) treatments. They found that sucrose and CK treatments could overcome SL-inhibited bud release. Thus, the authors established a very clear connection between SL, CK, and sucrose during early stage of bud growth and provided insights into the mechanism responsible for releasing axillary buds.

**Figure 1.**
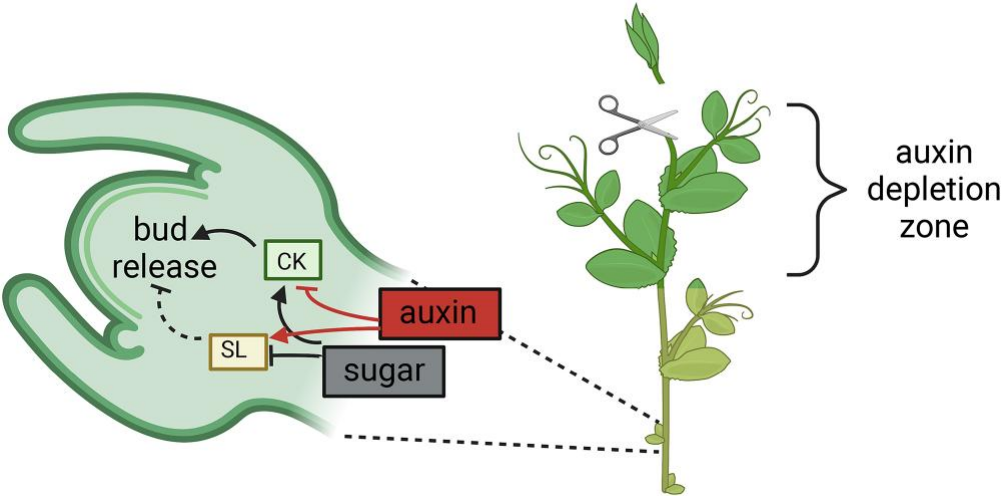
An overview of the interactions between sucrose and CK mediating bud release after apex decapitation. Following shoot apex removal, dormant buds outside the auxin-depletion zone rapidly accumulate sugar and CK, which lead to a decrease in SL response and subsequent bud release. The suppression of CK by auxin is lost after decapitation. Dashed lines indicate regulations that are lost during bud release process. Solid lines indicate regulation presents during bud release. Blunt end indicates downregulation and arrow end indicates upregulation. Created with BioRender.com.

GA is also known to regulate bud release. In this study, Cao and colleagues examined the response of dormant and released buds to GA treatments. Notably, they observed that GA treatment did not release bud growth during at least 3 d after treatment. By using a GA biosynthesis deficient pea mutant (*le)*, the researchers observed that buds in *le* mutants grew significantly slower than WT-decapitated plants. The authors measured the endogenous GA levels in different internodes and nodes of pea at various time points after decapitation. In the lower node, located outside the auxin-depletion zone (Fig. 1), GA levels increased 4 h after initial bud growth, indicating that GA is unlikely to be involved in triggering bud release. In contrast, in the stem region near the removed apex, GA levels were already decreased 3 h after decapitation. To establish a better understanding between the interplay of auxin and GA, the investigators quantified GA and auxin levels at different time points in upper and lower internodes and in the node outside the auxin-depletion zone. Notably, they observed a clear correlation between GA and auxin in the node, but not in any of the internodes. Finally, the authors investigated whether GA acts downstream of auxin during bud outgrowth by treating the bud located outside the auxin-depletion zone with GA and auxin inhibitors and measuring its growth after decapitation. Interestingly, the results showed that GA reversed the effect of auxin inhibitors in the decapitation-induced bud growth, supporting the idea that GA acts downstream of auxin.

Altogether, the study conducted by Cao et al. (2023) uncovered relevant information about the regulation of bud release and growth. Most studies on bud growth focus on understanding how 1 or 2 signals control bud development. This work provided evidence of the interconnectivity among the main signals controlling bud growth (CK, auxin, GA, and sucrose). In addition, the authors provided compelling evidence supporting the idea of sucrose as the key signal that triggers a whole set of hormonal changes to control bud release.
